# Comparing and Contrasting Traditional Membrane Bioreactor Models with Novel Ones Based on Time Series Analysis

**DOI:** 10.3390/membranes3010016

**Published:** 2013-02-06

**Authors:** Parneet Paul

**Affiliations:** School of Engineering and Design, Brunel University, Uxbridge, Middlesex UB8 3PH, UK; E-Mail: parneet.paul@brunel.ac.uk; Tel.: +44-1895-265-435; Fax: +44-1895-274-000

**Keywords:** wastewater treatment, membrane bioreactor, modelling, time series analysis

## Abstract

The computer modelling and simulation of wastewater treatment plant and their specific technologies, such as membrane bioreactors (MBRs), are becoming increasingly useful to consultant engineers when designing, upgrading, retrofitting, operating and controlling these plant. This research uses traditional phenomenological mechanistic models based on MBR filtration and biochemical processes to measure the effectiveness of alternative and novel time series models based upon input–output system identification methods. Both model types are calibrated and validated using similar plant layouts and data sets derived for this purpose. Results prove that although both approaches have their advantages, they also have specific disadvantages as well. In conclusion, the MBR plant designer and/or operator who wishes to use good quality, calibrated models to gain a better understanding of their process, should carefully consider which model type is selected based upon on what their initial modelling objectives are. Each situation usually proves unique.

## 1. Phenomenological Model-Duclos-Orsello (2006)

A phenomenological dead-end filtration model [1] was used in this study that depicts the three main fouling mechanisms occurring on membranes, namely cake build-up, complete pore blocking, and pore constriction, as originally described in Hermia [2] under constant trans-membrane pressure (TMP) operation. In this model, Duclos-Orsello [1] splits the total flow, *Q_t_*, through the membrane into flow through the unblocked membrane surface area and flow through the blocked membrane surface area as shown in Equation (1). Hence the first algebraic term relates to the unblocked flow whilst the second integral term relates to blocked flow:

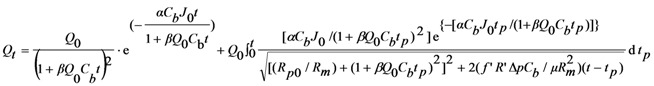
(1)


This initial model was extensively modified and added to by Paul *et al.* [3], so that it could be used to model a variety of real life membrane bioreactor (MBR) configurations. Modifications and add-ons to this basic model included: alteration so that it could be used for varying flux and varying TMP operations; inclusion of a backwash mode; it described pore constriction (*i.e.*, irreversible fouling) in relation to the concentration of soluble microbial product (SMP) in the liquor (with SMP being the key agent thought to determine membrane fouling [4]); and, it could be used in a cross flow scenario by the addition of scouring terms in the model formulation. Using data collected from a pilot membrane filtration unit, this modified deterministic model was calibrated and validated in Matlab^©^.

### Disadvantages of Using Phenomenological Membrane Fouling Models for Plant Design, Operation and Control

Most current researchers model the membrane fouling process using a phenomenological mechanistic approach that obeys the fundamental laws of physics. This is the traditional approach used to model MBR systems that treat wastewater. However it has been found that it does suffer from the following disadvantages:

− Membrane fouling is in reality highly complex and currently poorly understood as a process. Hence any mechanistic fouling model, either simple or complex, cannot hope to adequately address all aspects involved in the fouling procedure;
− Usually a mechanistic fouling model needs to be made bespoke for each individual filtration system so that it accurately depicts the specific hydrodynamics of the process and the membrane operational regime;
− These models are normally highly dimensional and contain several parameters requiring determination by real life plant data sets (e.g., flux stepping trials, extended specialist laboratory experiments, *etc.*);
− Parameter estimation and optimisation require expert knowledge and proves to be complex as most models of this type are over-parameterised with too many degrees of freedom;
− For many applications insufficient quality data is usually available to allow a full model calibration and validation, and thus any verified model is not accurate for every situation;
− The general application of such complex models means their take up for process control and the development of future operational strategies will always prove limited [5].

In a bid to overcome the distinct disadvantages of a traditional mechanistic approach, it has been suggested that a non-traditional approach can be used to describe the membrane filtration and fouling process for a MBR system. The non-traditional approach which was used in this study is based on time series system identification methods [6]. At its simplest, it uses the plant data set itself to determine that best fit model from a range of standard numerical model structures (e.g., autoregressive, state-space, sub-space, *etc.*), that are described in greater detail in the next section.

## 2. Using Time Series System Identification Methods to Create Input-Output (IO) Models as a Possible Non-Traditional Alternative

System identification is an iterative process in which models with different structures are identified from data, and the individual model performance compared. The normal start point is by estimating the parameters of very simple model structures. If the performance still proves poor, then the model structure is gradually increased in complexity. Ultimately the simplest of all model structures tested is eventually selected that best describes the dynamics of the system under scrutiny. In this iterative process, which can be automated, the system identification procedure commences by initially using linear continuous input-output (IO) model structures. This followed by using more complex non-linear structures. The best fit structure is then chosen as the optimal model formulation.

Historically speaking the first real instance of using a times series approach to analyse wastewater treatment data was carried out by Berthouex *et al.* [7], and later on by Debelak and Sims [8], and Christodoulatos and Vaccari [9]. Following these initial studies into using autoregressive time series methods for analysing wastewater treatment processes, they did seem to lose their appeal in the late 1990s and other artificial intelligence (AI) methodologies became in vogue instead. This loss of appeal was probably less to do with their predictive capacity and more to do with the increasing popularity and familiarity with AI methodologies by researchers over a wide range of differing academic disciplines.

In this study a linear continuous IO state-space model structure is tested using the supplied times series data. The state space model structure is a good choice for quick estimation because it requires only two parameters, namely the model order and one or more input delays. These model formulations are usually solved using iterative optimisation techniques and algorithms like the least squares method. However, this requires a lot of computing power and they are prone to inherent inaccuracies. A much more attractive model formulation is the sub-space one which does not need to be solved using iterative optimisation techniques and algorithms, but by only using algebraic calculations [10]. This means the sub-space model formulation is a very powerful version of the state-space one that uses only a single-shot solving procedure with improved accuracy. Hence this sub-space method was also tested under this study for comparative purposes.

## 3. Calibration and Validation of Both Model Types

### 3.1. Pilot Membrane Filtration Unit

**Figure 1:**
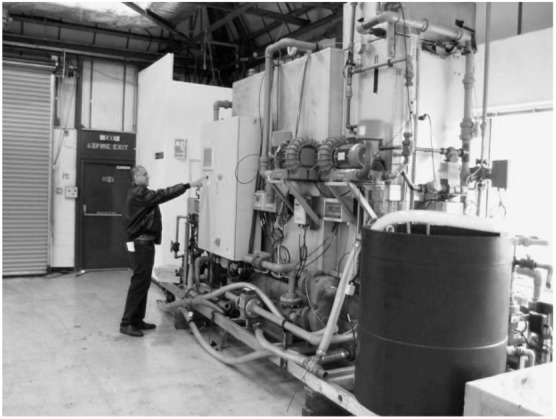
ITT Sanitaire membrane filtration unit (depicted with bioreactor).

Both fouling model types have been tested on data obtained from flux stepping tests performed on an ITT Sanitaire Ltd. (Colwick, Nottingham, UK) pilot membrane filtration unit depicted in Figure 1. This unit treated tertiary effluent from Cardiff’s sequence batch reactor (SBR) wastewater treatment plant, and its basic operational information is described in Table 1 below.

**Table 1:** Operational data for pilot membrane filtration unit.

ITT Sanitaire membrane filtration unit (without bioreactor)	
Membrane type and area	Horizontal “Kolon” fibres; PVDF 0.1 μm pore size; 20 m^2^
Feed flow; permeate flow; backwash	1 to 2.4 m^3^/h; 0.6 to 1 m^3^/h; 1.2 to 1.8 m^3^/h
Backwash interval & duration	Every 4 min with 30 s ON
TMP	300 to 500 mbar
Aeration rate	13 Nm^3^/h from coarse bubble tube diffuser
Cleaning regime	hypochlorite dosed 4 times daily into permeate tank
Feed flow biological data	COD concentration 50 mg O_2_/L; TSS concentration 25 mg/L
Indicative feed flow SMP data	Measured glucose concentration 5 mg/L; measured protein concentration 100 mg/L

### 3.2. Model Simulation—Results for Duclos-Orsello (2006) Traditional Approach

**Figure 2:**
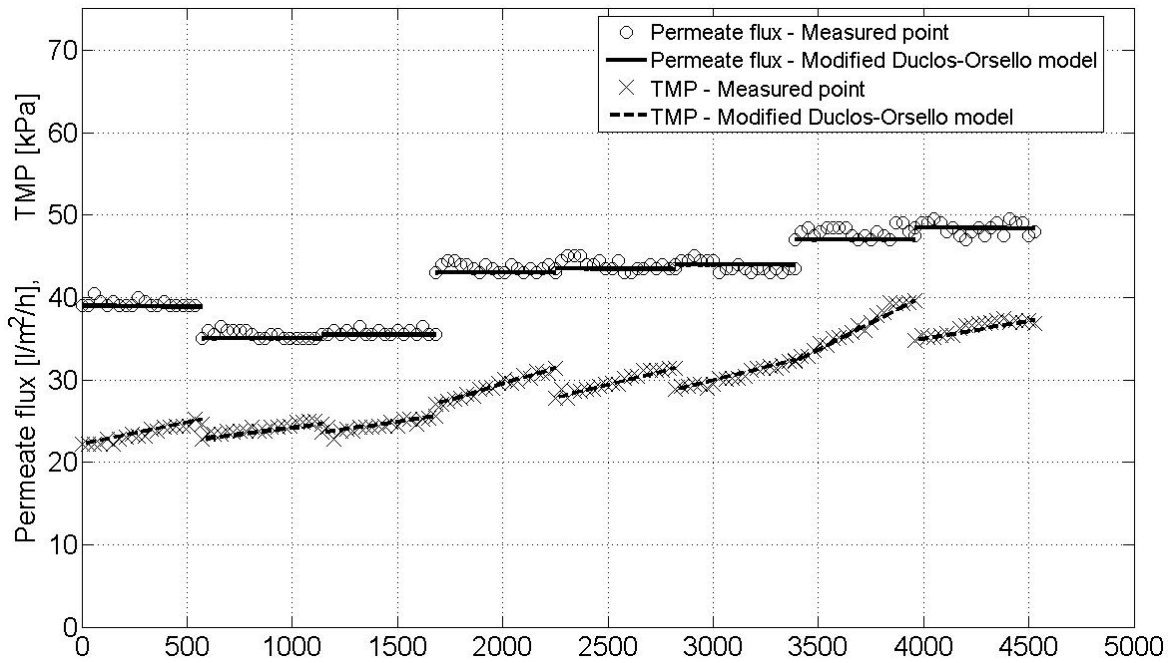
Modified phenomenological model—best model fit for 8 flux steps.

After various assumptions and simplifications of the plant data, the eight best flux steps were used to test the modified phenomenological model. Figure 2 shows the result obtained when using the calculated optimal parameter sets for the best eight flux steps for this pilot unit. The model fit is extremely good.

### 3.3. Model Simulation—Results for Non-Traditional Approach Using IO Models

After various assumptions and simplifications of the plant data, the eight best flux steps were used to test the proposed multi-input single output (MISO) model structure. As the plant layout for this unit is very simple with no bioreactor to complicate matters, the selected MISO model structure should give a very high degree of accuracy. In this case the permeate flux, the measured SMP levels, and the measured bulk mixed liquor concentration into the membrane were used as variables in the input model vector with the TMP being the single variable in the output model vector. Firstly an IO model based on a standard iterative state-space formulation was tested. This was followed by using a quicker single-shot algebraic sub-space method as a comparison. Results are described below.

#### 3.3.1. Best Fit for 8 Flux Steps for MISO Normal State-Space Model

**Figure 3:**
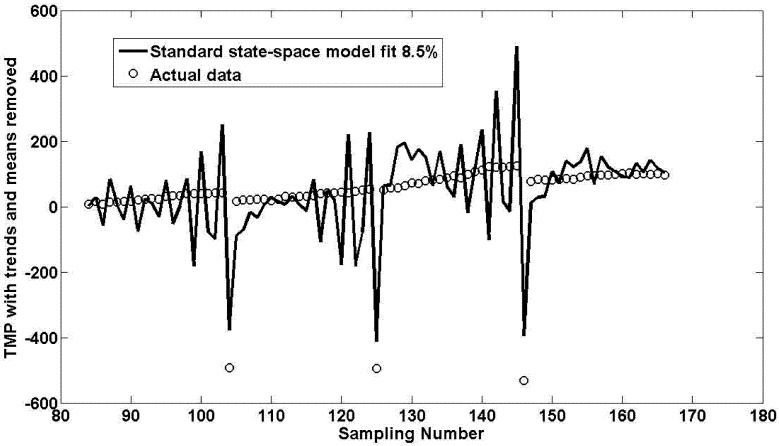
Best model fit for 8 flux steps (4 for validation) for standard state-space formulation.

Figure 3 shows the result obtained when using the calculated optimal parameter sets for the best eight flux steps for this pilot unit. The model fit is extremely poor.

#### 3.3.2. Best Fit for 8 Flux Steps for MISO Sub-Space Model

**Figure 4:**
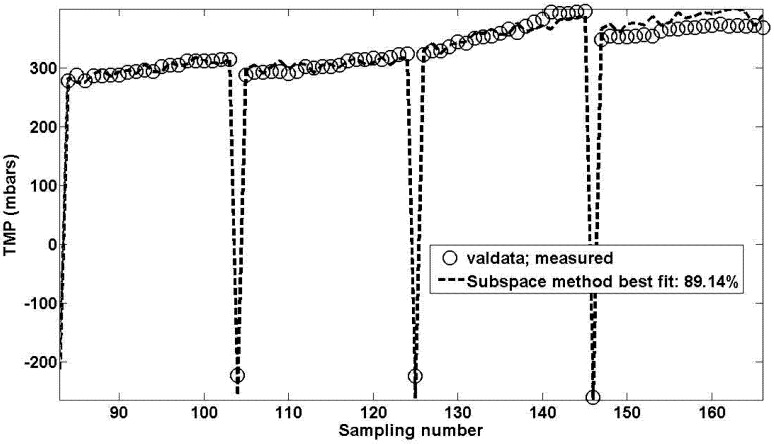
Best model fit for 8 flux steps (4 for validation) for sub-space method.

Figure 4 shows the result obtained when using the calculated optimal parameter sets for the best eight flux steps for this pilot unit. The model fit is very good.

### 3.4. Discussion of Model Simulation Results

The standard state-space formulation, whose equations are not given here for the sake of brevity, gave a workable fit, albeit not a very good one of 8.5% as shown in Figure 3. However, the shape and direction of the fit is correct even though the simulated data is prone to gradually attenuating fluctuations around a mean point. This poor fit could be attributed to the regular backwash events that cause a sudden large negative drop in the TMP that the simulated model in this case is unable to cope with.

When this MISO model structure is run as a subspace formulation, the best fit is for a 6th order model with an algorithm block size of 4. This fit is carried out by using the last four flux stepping cycles as the validation data set. Again for simplicities sake, the subspace model equations are not given here. The result as shown in Figure 4 depicts an excellent fit amounting to 89.14%. The shape of the fit is extremely good and is in the right direction (*i.e.*, TMP increases with time), thus validating the use of additional input biochemical data (e.g., SMP and mixed liquor concentration levels) to improve the overall model fit.

## 4. Conclusions

Overall it is clear that the phenomenological model performed very well even though it took a considerable time to be developed into a useful format, and the model had to be calibrated using complex genetic algorithm procedures. Conversely, the subspace method gave consistent results for the IO models used, and was very easy to set up and calibrate.

It initially looks like this novel approach has many advantages over traditional mechanistic models while giving comparable results for some IO structures. Early simulation results described in this study prove this, especially for subspace methods. However these methods can prove very fragile and prone to crashing. Additionally a comprehensive “Model Conceptualisation Procedure” is required to tie it into reality which needs expert know-how to set up. They also require very large data sets to produce accurate formulations, and these linear models are only useful around a very narrow operating range or operating point. Non-linear model versions can improve the predictive accuracy but are even more fragile.

In conclusion, it may prove advantageous to use these methods for model prediction under most circumstances apart from the following instances:

− Not for design of new plant (particularly for processes with long time constants), and the biological operation of plant (*i.e.*, off-line measurements).
− No good as research tools to investigate membrane fouling. Cannot predict one-off fouling events, only generalised scenarios.

The situation, in which they may particularly prove themselves superior to traditional model structures, is for model predictive control (possibly in real time) for processes with very short time constants (*i.e.*, rapidly changing flux/TMP data). However they would need constant automated updating of historical data sets using on-line sensors. In conclusion further research is required using longer historical data sets to definitively ascertain whether this non-traditional modelling approach can be further developed and improved upon.

## Nomenclature

*C_b_*: Bulk concentration (g/L)
*f*: Fractional amount of total foulant contributing to deposit growth
*J*_0_: Initial flux rate of clean membrane (m/s)
*Q_t_*: Total volumetric flow rate (m^3^/s)
*Q*_0_: Initial volumetric flow rate (m^3^/s)
*R_m_*: Resistance of the clean membrane (m^−1^)
*R*_*p*_0__: Original resistance of the deposit (m^−1^)
*R*: Specific protein layer resistance (m/kg)
*t*: Filtration time (s)
*t_p_*: Filtration time after initial membrane blocking occurs (s)
∆*p*: Constant total membrane pressure (Pa)

## Greek Letters

*α*: Pore blockage parameter (m^2^/kg)
*β*: Pore constriction parameter (kg)
